# Territory-rooted curiosity and public service: the legacy of dr. Paul Pachas

**DOI:** 10.17843/rpmesp.2025.424.15711

**Published:** 2025-11-17

**Authors:** César Cabezas

**Affiliations:** 1 Instituto Nacional de Salud. Lima, Peru. Instituto Nacional de Salud Lima Peru

In Peru, the vocation for public health is often born before the classroom: in the childhood that asks why neighbors get sick, in the adolescent who follows the tracks of what happens in their environment, and in the student who discovers that curiosity can be organized into a method. Dr. Paul Pachas (Figures 1 and 2) embodied that journey: he turned the territory into a classroom, the question into a design, and the evidence into a response organized with other actors. He made field epidemiology a profession of rigor and service, reminding us that the curves on a graph are not just lines, but the representation of biographies at risk.

**Figure 1:**
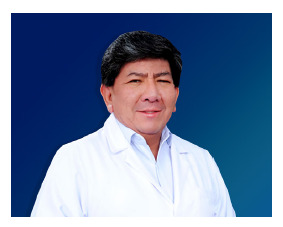
Dr. Paul Pachas, epidemiologist physician at the National Institute of Health.

**Figure 2:**
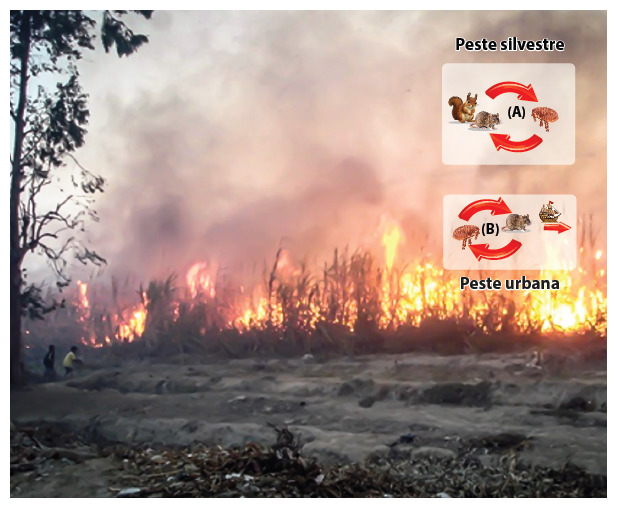
A vocation for public health often begins before the classroom, in real-life situations: The case of the plague in Peru, as we learned from Dr. Paul Pachass ^(^^5^.

## Training, Career, and Professional Maturation

A San Fernando physician from the National University of San Marcos, he oriented his development toward public health and field epidemiology ^1^, with special attention to vector-borne and zoonotic diseases. He actively participated in the surveillance, investigation, and control of outbreaks in Andean and Amazonian regions, and collaborated with teams from the Regional Health Directorates (DIRESA, Spanish acronym), the Ministry of Health (MINSA, Spanish acronym), and the National Institute of Health (INS, Spanish acronym). His profile integrated clinical practice and epidemiology, incorporating operational tools that strengthened the circuits between surveillance, laboratory, and an adequate and timely health response.

Among the multiple milestones of his valuable contributions, his work against Carrión’s disease (bartonellosis), leptospirosis, plague, sylvatic rabies, rickettsiosis, dengue, among others, stands out; the promotion of cost-effective preventive measures in high-risk populations; and his permanent insistence that every health decision be supported by quality surveillance, timely analysis, and transparent communication. Beyond the number of publications, his mark remained in implementation ^2^: transforming processes to protect lives; in short, the “solucionática” (solution-oriented approach), as taught by the Master Joaquín Cornejo Ubilluz.

It is necessary to highlight a Peruvian tradition made of identity, curiosity, and commitment. In this context, the biography of Paúl Pachas, born in San Vicente de Cañete, dialogues with a constellation of physicians who linked their identity to the territory and turned it into a scientific and moral driving force: Daniel A. Carrión of Cerro in Cerro de Pasco, Cayetano Heredia in Catacaos, Manuel Núñez Butrón in Puno, and Julio C. Tello, physician and archaeologist, in Huarochirí. In all of them, curiosity and commitment were inseparable: the question was never exhausted in theory, but translated into an organized response. That is the thread that Pachas sustained with exemplary sobriety.

## Dr. Paul Pachas also leaves three lessons for the present

First: the territory as a permanent classroom, today under a *One Health* approach ^3^, where humans, animals, and the environment interact dynamically.

Second: research as a service, operational questions that improve diagnostic circuits and strengthen the response to outbreaks.

Third: mentorship as a silent public policy, forming and leaving installed capacities that multiply the protection of the population ^4^.

Finally, if we want to move from memory to agenda, six feasible lines of action can be identified that Dr. Paul Pachas leaves us as challenges:

a) Territorial breeding grounds (seedbeds) from school and undergraduate level.

b) Early immersions with applied research objectives.

c) Articulated SERUMS-postgraduate pathways, with credits and scholarships for field epidemiology.

d) Agile funding for priority operational questions.

e) Mentorships and intergenerational networks INS-universities-DIRESA.

f) Metrics that value relevance and implementation, in addition to publications that demonstrate impact.

## The Ethics of Service

Those who worked with him often summarize his legacy in a simple conviction: no one should leave this world without having won, at least one battle for others. Paul won many, without fanfare and with the satisfaction of duty fulfilled. May his example commit us, as a community, to align incentives so that every girl and boy who today asks “Why is this happening in my community?” finds teachers, paths, and resources to turn that question into life-saving science, as Dr. Paul Pachas taught us.
