# Supplementary material for: Territory-rooted curiosity and public service: the legacy of dr. Paul Pachas
Source: Rev Peru Med Exp Salud Publica. 2025 Nov 17;42(4):347–8. doi: 10.17843/rpmesp.2025.424.15711 (PMC12879976; doi:10.17843/rpmesp.2025.424.15711)
Supplement: Translation

# Translation: Curiosidad enraizada en el territorio y servicio público: el legado del dr. Paul pachas

**Authors:** César Cabezas

**Affiliations:** 1 Instituto Nacional de Salud. Lima, Perú.

En el Perú, la vocación por la salud pública suele nacer antes del aula: en la infancia que pregunta por qué enferman los vecinos, en el adolescente que sigue las pistas de lo que ocurre en su entorno y en el estudiante quien descubre que la curiosidad puede organizarse en método. El Dr. Paul Pachas (Figuras 1 y 2) encarnó ese trayecto: convirtió el territorio en aula, la pregunta en diseño y la evidencia en respuesta organizada con otros actores. Hizo de la epidemiología de campo un oficio de rigor y de servicio, recordándonos que las curvas de un gráfico no son solo líneas, sino la representación de biografías en riesgo.

**Figura 1:** Dr. Paul Pachas, médico epidemiólogo del Instituto Nacional de Salud.

**Figura 2:** La vocación por la salud pública suele nacer antes del aula, en la realidad: El caso de la peste en el Perú, como aprendimos del Dr. Paul Pachas ^(^^5^.

## Formación, trayectoria y maduración profesional

Médico Sanfernandino de la Universidad Nacional Mayor de San Marcos, orientó su desarrollo hacia la salud pública y la epidemiología de campo ^1^, con especial atención a enfermedades metaxénicas y zoonóticas. Participó activamente en la vigilancia, investigación y control de brotes en regiones andinas y amazónicas, y colaboró en equipos de las Direcciones Regiones de Salud (DIRESA), del Ministerio de Salud (MINSA) y del Instituto Nacional de Salud (INS). Su perfil integró la clínica y la epidemiología, incorporando herramientas operativas que fortalecieron los circuitos ente vigilancia, laboratorio y una respuesta sanitaria adecuada y oportuna.

Entre los múltiples hitos de sus valiosos aportes, destacan su trabajo frente a la enfermedad de Carrión (bartonelosis), leptospirosis, peste, rabia silvestre, rickettsiosis, dengue, entre otras; la promoción de medidas preventivas costo-efectivas en poblaciones de alto riesgo; y su permanente insistencia en que toda decisión sanitaria se sustente en vigilancia de calidad, análisis oportuno y comunicación transparente. Más allá del número de publicaciones, su huella quedo en la implementación ^2^: transformar procesos para proteger vidas; en pocas palabras, la «solucionática», como también enseñara el Maestro Joaquín Cornejo Ubilluz.

Es necesario destacar una tradición peruana hecha de identidad, curiosidad y compromiso. En ese contexto, la biografía de Paúl Pachas, nacido en San Vicente de Cañete, dialoga con una constelación de médicos que vincularon su identidad al territorio y lo convirtieron en motor científico y moral: Daniel A. Carrión de Cerro en Cerro de Pasco, Cayetano Heredia en Catacaos, Manuel Núñez Butrón en Puno y Julio C. Tello, médico y arqueólogo, en Huarochirí. En todos ellos, curiosidad y compromiso fueron inseparables: la pregunta nunca se agotó en la teoría, sino que se tradujo en respuesta organizada. Ese es el hilo que Pachas sostuvo con ejemplar sobriedad.

## El Dr. Paul Pachas deja también tres lecciones para el presente

Primera: el territorio como aula permanente, hoy bajo una mirada *One Health*^3^, donde humanos, animales y ambiente interactúan dinámicamente.

Segunda: la investigación como servicio, preguntas operacionales que mejoran circuitos diagnósticos y fortalecen la respuesta a brotes.

Tercera: la mentoría como política pública silenciosa, formar y dejar capacidades instaladas que multiplican la protección de la población ^4^.

Finalmente, si queremos transitar de la memoria a la agenda, pueden identificarse seis líneas de acción factibles que nos deja como retos el Dr. Paul Pachas:

a) Semilleros territoriales desde la escuela y el pregrado.

b) Inmersiones tempranas con objetivos de investigación aplicada.

c) Trayectos articulados SERUMS-posgrado, con créditos y becas para la epidemiología de campo.

d) Financiamiento ágil para preguntas operacionales prioritarias.

e) Mentorías y redes intergeneracionales INS-universidades-DIRESA.

f) Métricas que valoren pertinencia e implementación, además de publicaciones que evidencien impacto.

## Ética del servicio

Quienes trabajaron con él suelen resumir su legado en una convicción sencilla: nadie debería irse de este mundo sin haber ganado, al menos una batalla por los demás. Paúl ganó muchas, sin estridencias y con la del deber cumplido. Que su ejemplo nos comprometa, como comunidad, a alinear incentivos para que cada niña y niño que hoy pregunta «¿por qué pasa esto en mi comunidad?» encuentre maestros, caminos y recursos para convertir esa pregunta en ciencia que salva vidas, como nos enseñó el Dr. Paul Pachas.
